# Ultrathin Carbon with Interspersed Graphene/Fullerene-like Nanostructures: A Durable Protective Overcoat for High Density Magnetic Storage

**DOI:** 10.1038/srep11607

**Published:** 2015-06-25

**Authors:** Neeraj Dwivedi, Nalam Satyanarayana, Reuben J. Yeo, Hai Xu, Kian Ping Loh, Sudhiranjan Tripathy, Charanjit S. Bhatia

**Affiliations:** 1 Department of Electrical and Computer Engineering, National University of Singapore, 117583 Singapore; 2 Graphene Research Centre and Department of Chemistry, National University of Singapore, 117543 Singapore; 3 Institute of Materials Research and Engineering (IMRE), A*STAR (Agency for Science, Technology, and Research), 3 Research Link, 117602 Singapore

## Abstract

One of the key issues for future hard disk drive technology is to design and develop ultrathin (<2 nm) overcoats with excellent wear- and corrosion protection and high thermal stability. Forming carbon overcoats (COCs) having interspersed nanostructures by the filtered cathodic vacuum arc (FCVA) process can be an effective approach to achieve the desired target. In this work, by employing a novel bi-level surface modification approach using FCVA, the formation of a high sp^3^ bonded ultrathin (~1.7 nm) amorphous carbon overcoat with interspersed graphene/fullerene-like nanostructures, grown on magnetic hard disk media, is reported. The in-depth spectroscopic and microscopic analyses by high resolution transmission electron microscopy, scanning tunneling microscopy, time-of-flight secondary ion mass spectrometry, and Raman spectroscopy support the observed findings. Despite a reduction of ~37 % in COC thickness, the FCVA-processed thinner COC (~1.7 nm) shows promising functional performance in terms of lower coefficient of friction (~0.25), higher wear resistance, lower surface energy, excellent hydrophobicity and similar/better oxidation corrosion resistance than current commercial COCs of thickness ~2.7 nm. The surface and tribological properties of FCVA-deposited COC was further improved after deposition of lubricant layer.

With the growing usage and demand for digital data storage in recent years, the magnetic hard disk drive (HDD) industry is working towards finding solutions to achieve higher data storage densities. For HDDs, the target is to achieve an areal density of 4 Tb/in^2^ by 2020, which is a fourfold increase from today’s areal density of 1 Tb/in^2^ 1. One of the essential requirements to achieve the target is the reduction of head media spacing (HMS) to ~5 nm^1^. Among the various factors that contribute to HMS, the media overcoat thickness comprises the major part. Hence, the overcoat thickness should be reduced from ~2.5 nm for 1 Tb/in^2^ to ~1.6 nm for 4 Tb/in^2^ without compromising its functional performance^1^. Owing to promising protective characteristics, ultrathin carbon overcoats (COCs) are used on hard disk media to protect them from corrosion and mechanical wear. The sp^3^ carbon bonding is the key characteristic of the COC which contributes to improvement of its corrosion- and wear resistance^2^,^3^,^4^. However, lowering the thickness of conventional COCs deposited by plasma enhanced chemical vapor deposition (PECVD) or sputtering can introduce unavoidable tribological and corrosion issues, particularly at the thickness <2 nm^3^,^4^,^5^,^6^. This has spurred immense interest to search and explore alternative processes to tailor the microstructure of COCs such that the desirable functional performance can be realized at a thickness of <2 nm for future media overcoats.

Filtered cathodic vacuum arc (FCVA) has the ability to deposit continuous, dense and high sp^3^ bonded carbon films at such low thickness levels^3^,^6^,^7^. Moreover, recent studies have shown that FCVA-grown non-hydrogenated COCs possesses higher thermal stability than PECVD-grown hydrogenated carbon (CH_x_) overcoats, which may be due to the presence of high sp^3^ carbon bonding and the absence of hydrogen in FCVA-grown COCs^8^,^9^,^10^. This indicates that FCVA-processed COCs have great potential to be used in future magnetic recording technologies such as heat assisted magnetic recording (HAMR).

Advanced carbon nanomaterials such as fullerene-like carbon and graphene nanostructures show remarkable properties such as high hardness, low friction, high elasticity, high wear- and corrosion resistance^11^,^12^,^13^. Hence, the inclusion of graphene/fullerene-like carbon nanostructures in a highly sp^3^ bonded amorphous carbon (a-C) matrix could be an effective way to further enhance the characteristics of carbon films particularly at ultrathin film levels (<2 nm) for future HAMR application. While the presence of high sp^3^ carbon bonding helps to acheive high thermal stability as well as high corrosion- and wear resistance, the presence of these graphene/fullerene-like carbon nanostructures can contribute to further improvement of the wear- and corrosion resistance of the COC as well as to attain low friction. Thus, due to their synergetic effect the presence of graphene/fullerene carbon nanostructures in a highly sp^3^ bonded a-C ultrathin film may show improved functional performance for for future media overcoats. It has been proposed that the FCVA technique has the ability to produce a-C films embedded with graphene/fullerene-like nanocrystallites by using a high local gas pressure^14^,^15^,^16^. Amaratunga *et al.*^14^ and Alexandrou *et al.*^16^ confirmed the presence of graphene/fullerene-like nanocrystallites in amorphous carbon matrix by transmission electron microscopy (TEM) and electron energy loss spectroscopy (EELS), while Chhowalla *et al.*^15^ have employed mass spectrometry to investigate the generation of fullerene-like carbon structures in the plasma phase and later by TEM in the condensed state. By examining the functional performance, they found that a-C films with embedded fullerene-like and/or graphene-like nanocrystallites show high hardness, high elastic recovery, high wear resistance and low friction. Recently, Tembre *et al.*^17^ have also reported the synthesis of fullerene-like structures during the growth of cobalt carbon (Co-C) composite films by a pulsed arc process. Employing high C^+^ ion energy, high deposition temperature (above room temperature) and post deposition laser annealing the synthesis of amorphous carbon films with oriented graphitic-like structures by FCVA has also been demonstrated^18^,^19^,^20^. Apart from FCVA, amorphous carbon films with presence of fullerene-like nanostructures or fullerene-like carbon films can also be prepared by sputtering and PECVD^21^,^22^,^23^,^24^,^25^. However, in view of the advantages of FCVA-processed COCs for future hard disk drive application, the realization of such COCs by FCVA can be more interesting. So far, the literature related to the formation of graphene/fullerene-like and oriented nanostructures incorporated amorphous carbon films and their superior functional performances by either FCVA or sputtering and PECVD have involved thicker films ranging from ~25 nm to ~1500 nm^14^,^15^,^16^,^17^,^18^,^19^,^20^,^21^,^22^,^23^,^24^,^25^. However, to the best of our knowledge, there have not been any studies reported on the formation of ultrathin COCs (<2 nm) incorporated with these advanced carbon nanostructures.

In this work, through employing bi-level surface modification of media by carbon using FCVA, fabrication of <2 nm thick and high sp^3^ bonded COC with interspersed graphene/fullerene-like nanostructures is reported. In-depth characterizations by high resolution microscopic and spectroscopic techniques were employed to confirm and articulate the underlying phenomenon leading to the formation of such nanostructures in ultrathin (~1.7 nm) COC with superior functional performance. The comparison of the properties of the FCVA-processed COC with current thicker (~2.7 nm) commercial COC grown by PECVD clearly reflected the effectiveness of the FCVA process in developing the COC with low friction, high wear- and corrosion resistance and high hydrophobicity at thickness <2 nm. The perfluoropolyether (PFPE)-based lubricated (lube) was also applied on FCVA-deposited COC to compare the tribological and surface properties of media with our COC and lube and full commercial media with commercial COC and commercial lube.

## Results

**Figure 1:**
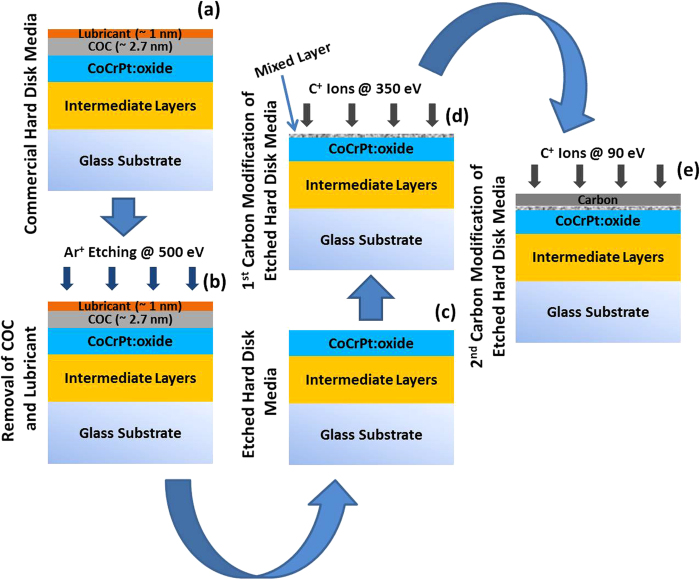
Schematic representation of the substrate and fabrication process of FCVA deposited carbon in sample S-3. (**a**) Current commercial hard disk media with magnetic layers, commercial COC and lubricant, (**b**) Ar^+^ ion etching of commercial media to remove the commercial COC and lube, (**c**) commercial media after removal of commercial COC and lubricant, (**d**) 1^st^ level carbon modification of etched commercial media at C^+^ ion energy of 350 eV using FCVA and (**e**) 2^nd^ level carbon modification of media at C^+^ ion energy of 90 eV using FCVA.

In this study, we included following 9 samples namely: plasma etched CoCrPt:oxide bare magnetic media without COC and without lube (S-1), specially prepared commercial media with commercial COC of thickness ~2.7 nm but no lube (S-2), media with ~1.7 nm COC deposited at 350 eV followed by 90 eV using FCVA (S-3), media with ~1.2 nm COC deposited at 20 eV using FCVA (S-4), media with ~1.6 nm COC deposited at 20 eV using FCVA (S-5), media with ~1.9 nm COC deposited at 90 eV followed by 50 eV using FCVA (S-6), plasma etched bare magnetic media without COC but with lube (S-7), full commercial media with ~2.7 nm commercial COC and ~1 nm commercial lube (S-8) and media with ~1.7 nm COC deposited at 350 eV followed by 90 eV using FCVA with ~1.4 nm lube. Out of these samples, the samples S-2, S-8, S-3 and S-9 lie at the core of this study and hence they are characterized and discussed in detail. To understand and explain the mechanism of the formation of unique nanostructures in the 350/90 eV grown FCVA-based COC in sample S-3, other low energy deposited FCVA-based COCs in samples S-4, S-5 and S-6 are also considered. Sample S-1 and S-7 are used for reference/comparison. A table containing the description of all these samples can be seen in supplementary information S1 and details of these samples can also be found in the experimental section. Figure 1a–e illustrates a schematic of the deposition process of sample S-3 (and other FCVA processed samples i.e. samples S-4, S-5 and S-6). The starting substrate used for the preparation of sample S-3 was a commercial hard disk media with its original commercial COC of thickness ~2.7 nm and lube of thickness ~1 nm. To deposit COC by FCVA, the commercial COC and lube were first removed from the substrates. This was done by *in situ* sputter etching of the substrates with Ar^+^ plasma at ion energy of ~500 eV for 300 s within the FCVA chamber and without breaking the vacuum of the chamber in order to avoid the surface oxidation of the media before FCVA treatment (Fig. 1a–c). The bi-level surface modification process was then performed by exposing the etched disks to first high energy C^+^ ions of 350 eV (Fig. 1d) followed by lower energy C^+^ ions of 90 eV (Fig. 1e).

**Figure 2:**
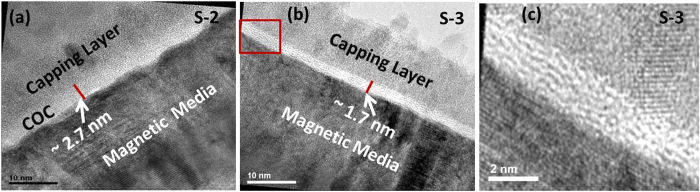
Cross-sectional HRTEM images. Samples (**a**) S-2 and (**b**) S-3. Figure (**c**) is the zoomed-in version of certain portion of sample S-3, which is indicated by red color rectangle in (**b**).

The thickness of the COCs in samples S-2 and S-3 were measured by cross-section high resolution transmission electron microscopy (HRTEM), as shown in Fig. 2a,b, respectively. Both of the images show CoCrPt-alloy based magnetic media, capping layer (titanium nitride), and the ultrathin COC in between the magnetic media and capping layers. The thickness of the commercial COC in sample S-2 was estimated to be 2.7 ± 0.1 nm, while the thickness of the COC in sample S-3 was measured to be 1.7 ± 0.1 nm. In addition, the HRTEM image of sample S-3 reveals an unusual nanolayered morphology of the COC, which does not appear in typical amorphous carbon films. This kind of ordering has been seen previously in graphene, carbon nanotubes (CNTs) and fullerene-like structures^14^,^21^,^26^. Figure 2c shows the zoomed-in image of a portion of the COC sample S-3, which is indicated by the red color rectangle in Fig. 2b. The HRTEM image reveals that the carbon structure contains curved basal planes, which results in crosslinking of the planes. These curved layer structures could be similar to that of fullerene-like structures as observed by Sjostrom *et al.*^21^ or fullerene-like nano-onions as detected by Hultman *et al.*^22^ The formation of fullerene-like composites has also been reported in case of the energetic deposition process in vacuum arc evaporation^14^,^15^,^16^. However, the curved layer structure was not observed in commercial COC (sample S-2).

### Scanning tunneling microscopy (STM)

**Figure 3:**
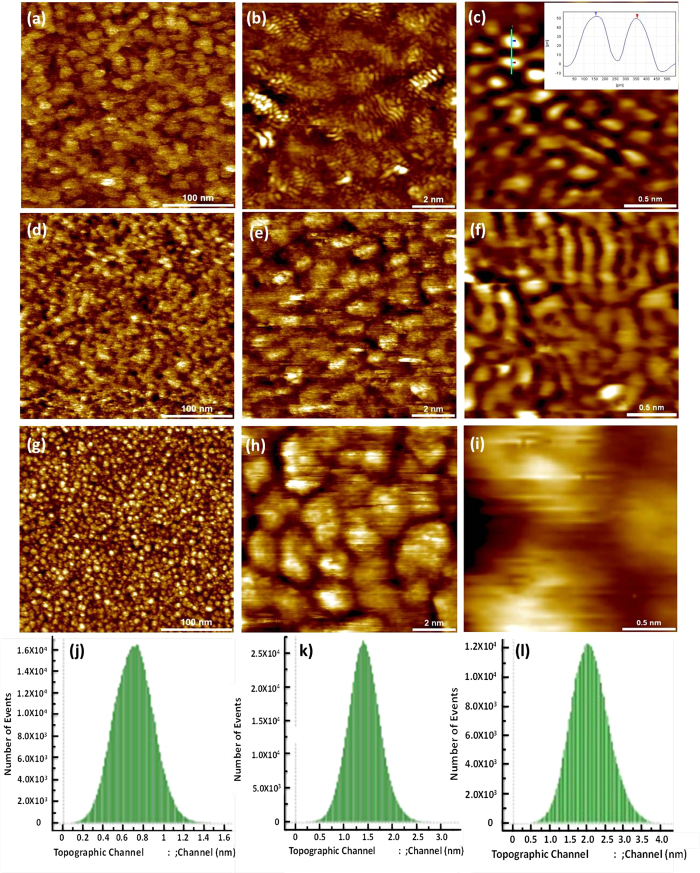
STM images. Samples S-3 [(**a–f**)] and S-2 [(**g–i**)] for topographical analysis. (**a**) The size of STM image in (**a**) is 300 × 300 nm^2^. (**b**) and (**c**) are the close-up STM images. (**d**), (**e**) and (**f**) are the STM topographical images of sample S-3 after annealing at 573 K in UHV. (**d**) The size of the STM image in (**d**) is 300 × 300 nm^2^. (**e**) and (**f**) are the close-up STM image. (**g**) The size of STM image in (**g**) is 300 × 300 nm^2^. (**h**) and (**i**) are the close-up STM images. (**j**), (**k**) and (**l**) are RMS histograms of the surfaces in (**a**), (**d**) and (**g**), respectively. All the STM images were recorded at similar conditions (I_t_ = 0.5–1 nA, V_g_ = 1 ~ 3 V).

To further analyze such carbon nanostrctures, scanning tunneling microscopy (STM) measurments were performed on sample S-2 and S-3 and the results are shown in Fig. 3. At first observation, the STM images of both of these COCs were found to show the formation of carbon nanostructures/nanoaggregates, which are different from as-deposited usual thicker carbon films that show amorphous carbon structures^27^. Although both types of COCs showed nanostructuring, their morphology and size of nanostructures were significantly different (Fig. 3a,g). From the STM image shown in Fig. 3a, the morphology of carbon in sample S-3 was found to be very flat with a root mean square (RMS) roughness only 0.2 nm. To gain further insight into the fine structure, we analyzed the surface of this sample in detail within an atomically resolved range as shown in Fig. 3b,c. The morphology of carbon in Fig. 3b appears to be similar to the morphology of defected graphene found on Cu (*see* supplementary information S2). This could lead to the possibility of the formation of graphene-like structures in the localized range. To further confirm the formation of such structures within the COC, the periodicity of carbon was analyzed. From Fig. 3c, it can be seen that the local periodicity is found to be 0.25 nm, which displays a graphene lattice. Recently, Hong *et al.*^28^ examined graphene by STM and observed a bipartite graphene honeycomb structure. In bipartite graphene, they realized one of the periods to be 0.246 nm (or ~0.25 nm) along the zigzag orientation and another period to be 0.426 nm along the armchair orientation. The zigzag orientation matches well with the period of carbon observed in sample S-3. Hence, the STM results indicated the formation of graphene-like nanostructures locally in S-3. However, long range periodicity was not observed in sample S-3. In Fig. 3c, the shape of the carbon network represents the curved graphene-like structure which is similar to those observed in fullerene-like onions deposited by the vacuum arc process^14^,^16^. The curved layer structure was also seen in the HRTEM analysis of sample S-3 (Fig. 2b,c). Hence, the overall structure of carbon in the sample S-3 can be said to consist of interspersed graphene/fullerene-like nanostructures within an amorphous matrix of carbon. Further, sample S-3 was annealed at 543 K for 30 min in ultra-high vacuum (UHV) to observe any temperature induced evolution in the morphology/topography of the carbon structure (Fig. 3d–f). A significant change in morphology of the carbon after annealing was observed with respect to its non-annealed counterpart. After annealing we did not observe an increase in the long range periodicity of the carbon structure. The STM images in the atomically resolved range (Fig. 3f) revealed the coalescence of carbon atoms after annealing, leading to destruction of local periodic structure. At the same time, the RMS roughness increased to 0.33 nm.

In order to reveal the uniqueness of the carbon structure found in sample S-3, we have also examined the surface of carbon in current commercial media sample S-2, which is shown in Fig. 3g–i. The structure of carbon in sample S-2 is found to be quite different from that of carbon incorporated with graphene/fullerene-like nanostructures as seen in sample S-3. The STM images of sample S-2 revealed the formation of carbon nanoaggregates (Fig. 3g). This sample was also analyzed in an atomically resolved range. From the atomically resolved images (Fig. 3h,i), we found that the morphology of carbon in this sample remained in a nanoaggregated form. The RMS roughness in sample S-2 was found to be 0.5 nm, which is comparatively higher than that of sample S-3. The RMS histograms of the surfaces in Fig. 3a,d,g are shown in Fig. 3j,k,l, respectively.

**Figure 4:**
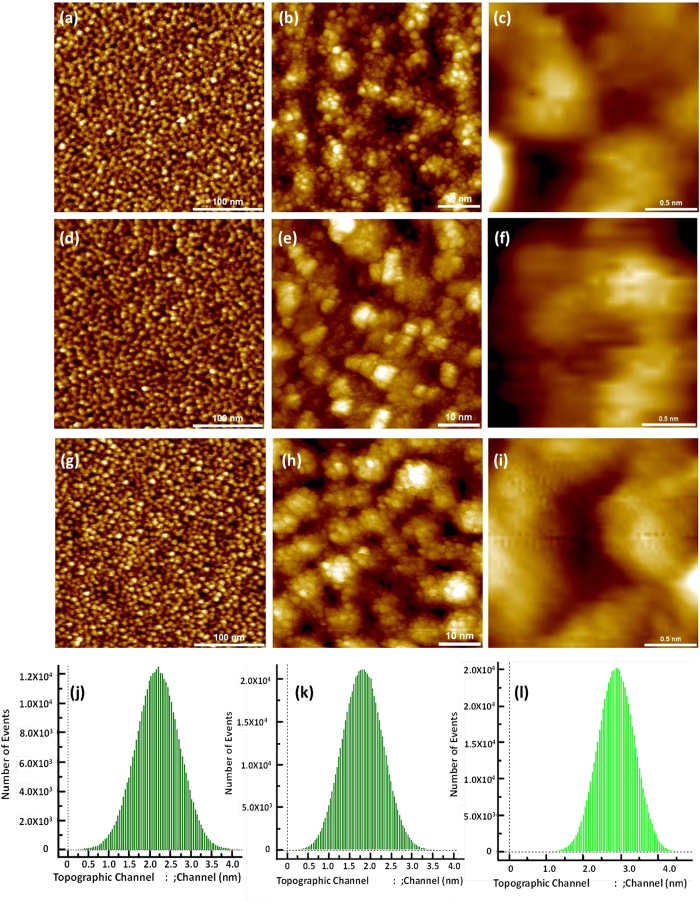
STM images. Ultrathin carbon films deposited at relatively lower ion energy of 20-25 eV [(**a–f**)] and at low energy bi-level surface modification of 90 eV followed by 50 eV (90/50 eV) [(**g–i**)] using FCVA. (**a**), (**b**) and (**c**) are the STM images for the carbon film with a thickness of 1.2 nm (sample S-4). (**d)**, (**e**) and (**f**) are the STM images for the carbon film with a thickness of 1.6 nm (sample S-5). (**g**), (**h**) and (**i**) are the STM images of the carbon film with a thickness of ~1.9 nm deposited using bi-level surface modification at 90/50 eV (sample S-6). (**j**), (**k**) and (**l**) are RMS histograms of the surfaces in (**a**), **(c**) and (**f**), respectively. All the STM images were recorded at similar conditions (I_t_ = 0.5–1 nA, V_g_ = 1 ~ 3 V).

To understand the role of energetics on the surface topography of FCVA-deposited carbon films, we have also deposited ultrathin carbon films on CoCrPt-alloy based media at a relatively lower ion energy of 20–25 eV and via moderate ion energy bi-level surface modification at 90 eV followed by 50 eV (90/50 eV) using FCVA. The thicknesses of the carbon films were kept as low as 1.2 ± 0.1 nm (sample S-4) and 1.6 ± 0.1 nm (sample S-5) for 20*–*25 eV grown carbon films, whereas a graded carbon film of thickness 1.9 ± 0.1 nm was prepared using C^+^ ion energies of 90 eV followed by 50 eV in a bi-level surface modification scheme (sample S-6). The thicknesses of these three carbon films were also estimated by HRTEM (not shown here). Figure 4a–c show the STM images of sample S-4, whereas Fig. 4d–f show the STM images of sample S-5. The STM images of both of these samples were found to be similar to those observed for sample S-2, which showed the formation of carbon nanoaggregates. Moreover, the RMS roughnesses of 0.53 nm and 0.48 nm were observed in samples with COC thicknesses of ~1.2 nm and ~1.6 nm, respectively, which were similar to the RMS roughness found in sample S-2 (0.5 nm). Meanwhile, the moderate ion energy bi-level surface modified film (90/50 eV, sample S-6) also revealed the formation of carbon nanoaggregates (Fig. 4g–i), which was found to be similar to that of commercial COC and COCs grown at low C^+^ ion energy (20–25 eV) by FCVA. Moreover, its RMS roughness, which was observed to be 0.49 nm, was also found to be similar to that of samples S-2, S-4 and S-5. The RMS histograms of the surfaces in Fig. 4a,d,g are shown in Fig. 4j,k,l, respectively.

### TOF-SIMS and Raman spectroscopy

**Figure 5:**
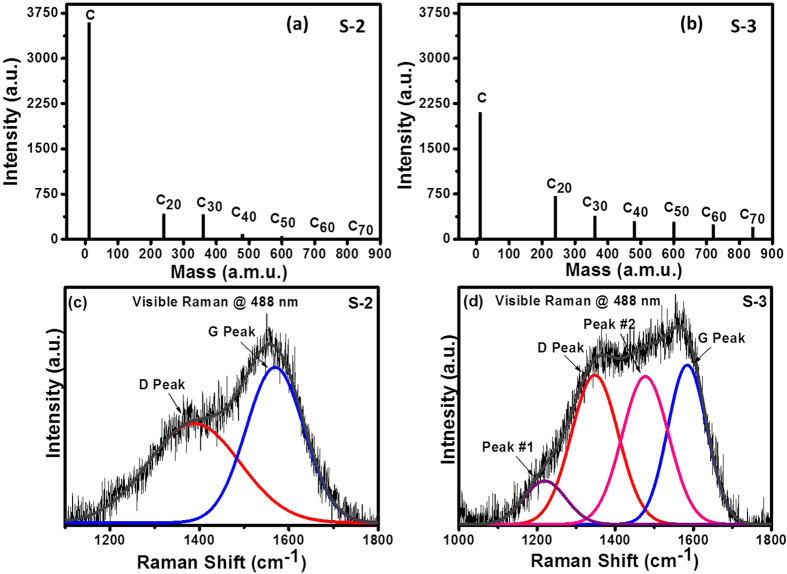
TOF-SIMS and Raman spectroscopy. Mass spectra of samples (**a**) S-2 and (**b**) S-3 showing different molecular structures of carbon. Visible Raman spectra of COC in samples (**c**) S-2 and (**d**) S-3.

Mass spectra by time-of-flight secondary ion mass spectrometry (TOF-SIMS) were recorded to understand the structure of carbon at the molecular level for samples S-2 and S-3. The TOF-SIMS mass spectra for these two samples are shown in Fig. 5a,b, respectively. The intensity scale for both of these samples was kept same to clearly visualize the intensity difference between the various species. The structure of a fullerene molecule (C_n_) is a caged structure of carbon, which possesses 12 pentagons and 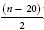
hexagons within the carbon network. Fused pentagons have a sp^3^-type of carbon network, whereas shared hexagons have a sp^2^-type of carbon network^26^. Hence, the inclusion and increase of hexagons in fullerene molecules leads to a structural transformation from sp^3^→sp^2^. This explains the C_20_ fullerene molecule (n = 20) possesses only pentagons (no hexagons) and shows a sp^3^-type of carbon network. However, when the hexagons in fullerene molecules are increased to 20 and 25 for C_60_ and C_70_, respectively, the structure transforms to sp^3^→sp^2^ carbon network. The C_60_ molecule reveals a sp^2.3^ bonding hybridization^29^. However, an exact sp^2^ hybridization can be attained by infinite fullerene, which is similar to a graphene plane with cyclic boundary conditions. Hence, the aim of recording the mass spectra is to find out the presence of fullerene-like structures in the deposited COCs. Although many molecular structures of fullerene have been described in the literature, only some of them are stable. Hence, we have limited our analysis to few species, namely C (12 amu), C_20_ (240 amu), C_30_ (360 amu), C_40_ (480 amu), C_50_ (600 amu), C_60_ (720 amu) and C_70_ (840 amu) as shown in Fig. 5a,b. The presence of C_60_ molecules is found to be very promising in sample S-3 though the presence of other molecules were also observed in both of these samples. The mass spectra of samples S-2 and S-3 show an intense peak at 12 amu, which is attributed to the neutral carbon. Interestingly, the mass spectra of these samples reveal the generation of a C_20_ peak. The intensity of the C_20_ peak was found to be ~1.5-1.6 times more intense in sample S-3 than in sample S-2, which is attributed to the presence of higher sp^3^ carbon bonding in sample S-3 as confirmed by XPS (discussed later). The generation of the C_20_ peak in the plasma of carbon by FCVA had been observed by Chhowalla *et al.*^15^ Paillard^29^ had also studied various small fullerene molecules (C_20_-C_32_) using a combination of mass spectroscopy (by TOF-SIMS) and deep UV Raman techniques, where the C_20_ peak was detected in the mass spectra. Apart from C_20_, C_30_ was also found to be present in both of these samples. However, the presence of C_40_, C_50_, C_60_, and C_70_ were negligible in sample S-2 although they were observed to be present in significant amounts in sample S-3.

It should be noted that except C_60_ and C_70_, all other forms of fullerene are not stable. C_60_ is the most stable form of fullerene followed by C_70_ fullerene. Hence, although sample S-3 showed many fullerene-like carbon species, the most exciting result is the generation of the C_60_ and C_70_ fullerene structures. However, C_60_ and C_70_ were found to be absent in the mass spectra of sample S-2. Considering the stability of fullerenes particularly in the condensed state, the formation of the fullerene-like structures could be judged based only on C_60_ and C_70_.

Raman spectroscopy is a widely used tool for structural characterization of carbon based materials. The main purpose for conducting Raman measurements is to gain greater insight into the structural ordering, which was seen in sample S-3. The visible Raman spectra of samples S-2 and S-3 are shown in Fig. 5c,d, respectively. The visible Raman spectrum of carbon in sample S-2 showed its signature D and G peaks. The D peak is due to the *A*_*1g*_ breathing mode of sp^2^ carbon atoms in rings. The G peak ascribes to in-plane bond stretching motion of pairs of sp^2^ carbon atoms in both aromatic rings and olefinic chains^30^. Surprisingly, the visible spectrum of carbon in sample S-3 was found to be quite different from that in sample S-2 in terms of band broadening. As a result of band broadening, the spectrum of carbon in sample S-3 could not be well fitted with two peaks. Further, usually the G peak of amorphous carbon films can be fitted using either Gaussian^31^,^32^,^33^,^34^, or Lorentzian^35^,^36^ or Breit-Wigner-Fano^30^,^37^ (BWF) functions, while the D peak can be fitted only by either Gaussian or Lorentzian functions. Though we could fit the G peak with the BWF function and the D peak with Lorentzian function for sample S-2 (*see* supplementary information S3), however, the G peak could not be well fitted with BWF function for sample S-3 due to the unusual shape of Raman spectrum of FCVA-deposited COC. The best fitting for sample S-3 was obtained when the Gaussian function was used. Hence, the Raman spectra fitted with two Gaussian components for sample S-2 and four Gaussian components for sample S-3 were discussed and compared. Many researchers had also observed similar Raman spectra of carbon and had fitted their spectra with more than two Gaussian components^38^,^39^,^40^. Along with the D and G peaks which are observed in the usual Raman spectra of amorphous carbon^41^, the visible Raman spectra of carbon in sample S-3 showed two additional peaks at 1220 cm^−1^ (peak #1) and 1477 cm^−1^ (peak #2). It is well stablished that fullerenes (C_60_ and C_70_) showed strong peak at ~1470 cm^−1^^42^. Kovacs *et al.*^28^, Wang *et al.*^39^, and Diaz *et al.*^43^ had also performed extensive Raman analysis on amorphous carbon films containing fullerene-like clusters and they had assigned peaks appearing in the range of 1470–1490 cm^−1^ to fullerene-like carbon. Hence, the peaks at ~1477 cm^−1^ in the visible Raman spectra of sample S-3 are assigned to fullerene-like carbon structures, which are localized distributed in an amorphous carbon matrix as evidenced by D and G peaks. On the other hand, pertaining to assignment of the peak in the range of 1150-1250 cm^−1^ to either sp^3^ phase of carbon^40^,^44^,^45^,^46^,^47^,^48^ or transpolyacetylene^49^ by the researchers, the observed peak at 1220 cm^−1^ could be accompanied to one of these structures.

Overall, based on HRTEM, STM, mass spectrometry and Raman analyses, the structure of COC in sample S-3 might be said to consist of amorphous carbon having some amount of interspersed graphene/fullerene-like nanostructures. On the other hand, sample S-2 revealed the presence of carbon nanoaggregates as evidenced by STM.

### Carbon hybridization, oxidation and corrosion analysis

**Figure 6:**
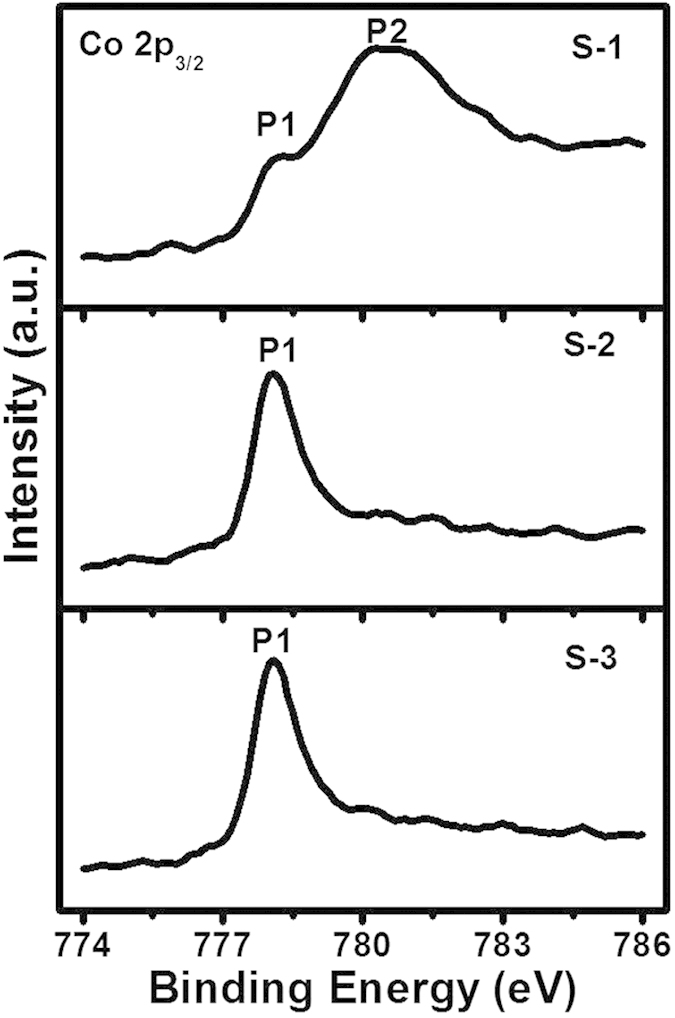
ARXPS analysis. Co 2p_3/2_ core level spectra of samples S-1, S-2 and S-3.

The carbon hybridization, and metallic and oxidation states of Co from samples S-1, S-2 and S-3 were analyzed by angle resolved X-ray photoelectron spectroscopy (ARXPS) at a photoelectron take-off angle of 65^o^ with respect to surface. Detailed ARXPS analysis on similar samples can be found in another paper by our group^50^. To discuss the structure-property relationship, we also presented some ARXPS results in supplementary information S4. Figure 6 shows the Co 2p_3/2_ core level spectra of these samples. Sample S-1 comprised two peaks – a minor and narrow peak close to 778.1 eV (P1) which corresponded to Co (Co in metallic state), and a broad and intense peak close to 780.4 eV (P2) which was assigned to Co-oxide. Comparing peak P1 with peak P2, we observed that peak P2 was dominant over peak P1, indicating that Co in S-1 was present mostly in its oxide states. However, peak P1 became more intense in samples S-2 and S-3 while peak P2 disappeared due to the introduction of the commercial and FCVA-processed COCs, which helped to reduce the oxidation of Co in media. While quantitatively analyzing the metallic and oxide contents of Co, we found that the thinner FCVA-processed COC in sample S-3 provided higher oxidation protection than the thicker commercial COC in sample S-2 (supplementary information S4). Moreover, electrochemical corrosion analysis of these samples (not included here) suggested that thinner FCVA-deposited COC had comparable corrosion resistance to thicker commercial COC, which is in agreement with our previous study carried out on similar samples^50^. The sp^3^C bonding is the key characteristic contributes to the higher density, higher oxidation/corrosion resistance and higher wear resistance properties of COCs. High sp^3^C bonding also provides high thermal stability, which is important for HAMR application. Hence, we calculated the sp^3^C bonding from the XPS C 1s core level spectra (supplementary information S4) and found them to be 33.0% and 40% in samples S-2 and S-3, respectively, which were also similar to the values obtained by Yeo *et al.*^50^

### Surface Energy analysis

**Figure 7:**
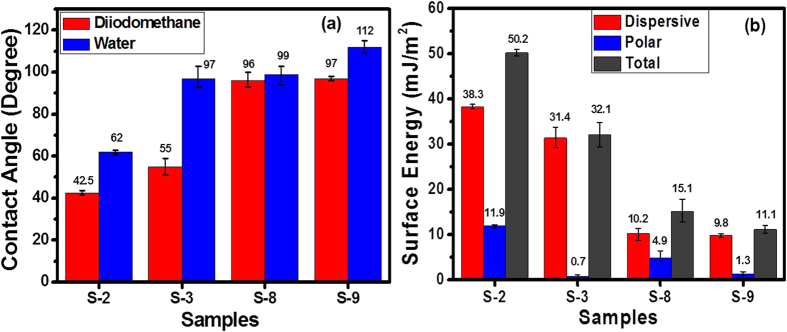
Analyses of surface energy and hydrophobicity. (**a**) water and diiodomethane contact angles and (**b**) dispersion, polar and total surface energy components for the media having commercial COC without and with lube and media having 350/90 eV FCVA-depsoited COC without and with lube.

Surface hydrophobicity is desirable, which might prevent media from corrosion and reduces the formation of water meniscii at the head-disk interface. Hence, contact angle measurements were performed on the samples of interest with two liquids, water and diiodomethane, to understand the interaction of water with overcoated media surfaces and to estimate the polar and dispersive components of surface energy. Figure 7a shows the bar chart of contact angles of water and diiodomethane for different samples. For non-lubricated samples, the water contact angle was found to be significantly higher while diiodomethane contact angle was observed to be slightly higher in sample S-3 than sample S-2. On the other hand, for lubricated samples the diiodomethane contact angle was almost similar in samples S-8 and S-9 but water contact angle was found to be higher in sample S-9 than S-8. This indicated that for non-lubricated samples, the FCVA deposited COC in sample S-3 seems to be more hydrophobic than sample S-2. After lubrication, the hydrophobicity was enhanced in both samples S-8 and S-9 as compared to their non-lubricated counterparts but sample S-9 showed relatively higher hydrophobic behaviour than sample S-8, as evidenced by the water contact angle. Further, the polar, dispersive and total surface energies of these samples were measured using standard theory (*see* supplementary information S5) and the results are shown in Fig. 7b. The results showed that the total surface energy was significantly lower in lubricated samples than their non-lubricated counterparts. For the non-lubricated samples, sample S-3 showed relatively lower total surface energy than sample S-2. The same trend was observed for lubricated samples and sample S-9 showed relatively lower total surface energy than sample S-8. It is interesting to note that the polar component of surface energy was found to be significantly lower in FCVA-deposited COC in both cases (without and with lube) than commercial COC without and with lube, respectively.

### Tribological properties

**Figure 8:**
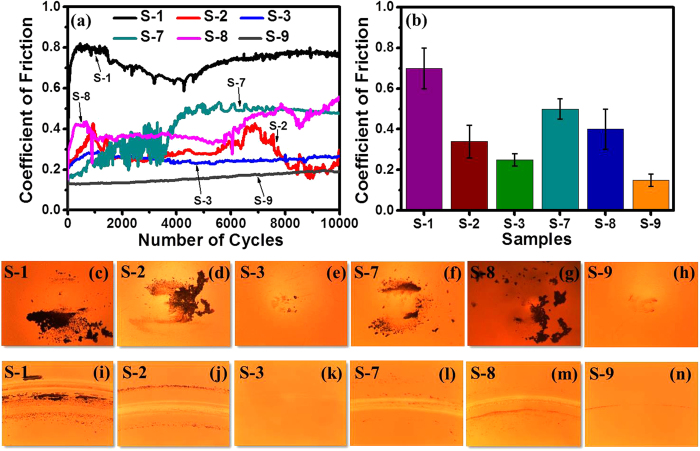
Tribological properties. (**a**) Frictional results (coefficient of friction versus number of cycles), (**b**) bar chart of the average COF values, (**c**–**h**) counterface ball images and (**i**–**n**) wear track images of samples S-1, S-2, S-3, S-7, S-8 and S-9 after the ball-on-disk tribological test.

The functional performance of these samples was examined using ball-on-disk tribological tests. The frictional results, ball images and wear track images of samples S-1, S-2, S-3, S-7, S-8 and S-9 after the ball-on-disk tribological tests are depicted in Fig. 8a–n. It can be seen from Fig. 8a,b that sample S-1 showed very high coefficient of friction (COF) of ~0.7, which is characteristic of bare magnetic media. Apart from high COF, significant material transfer to the ball (Fig. 8c) and a severe wear track (Fig. 8i) were also observed when sample S-1 was analyzed under an optical microscope, after completion of the test. The bare magnetic media, which possesses a stack of magnetic layers, is mechanically soft and wears easily when the counterface ball starts to slide against its surface. This suggests that bare media has very poor wear resistance. The introduction of commercial COC on media in sample S-2 was found to reduce the COF, though its value fluctuated throughout the test. The average value of the COF in sample S-2 was observed to be ~0.34. The ball and wear track images of sample S-2 (shown in Fig. 8d,j, respectively) were also analyzed to evaluate the tribological performance. As can be seen, there was a significant amount of debris on the ball and a visible wear track on the sample. In contrast, the FCVA-processed COC in sample S-3 exhibited remarkable tribological properties in terms of low (~0.25) and very stable COF until the end of the test, negligible debris on the ball and no visible wear track on the sample. The tribological results of this sample are promising as the thickness of this COC was only ~1.7 nm.

In order to examine the role of lubricant on the frictional and wear properties, ball-on-disk tribological tests were also carried out on lubricated samples S-7 and S-9 and full commercial media (sample S-8). Among samples S-7 to S-9, the sample S-7 showed a higher COF value but the value of this sample was lower than its non-lubricated counterpart (sample S-1). In addition, the optical images of sample S-7 showed slightly lower material transfer to the ball and a relatively less severe wear track as compared to sample S-1. On the other hand, commercial media sample S-8 with ~2.7 nm thick commercial COC and ~1 nm thick commercial lubricant showed almost similar behavior in terms of friction and wear to its non-lubricated counterpart (sample S-2). The most interesting results were observed in sample S-9. After introduction of lubricant on the FCVA-deposited COC, sample S-9 showed a very stable and low COF (<0.2) until the end of the test with negligible material transfer to the ball and a very minor wear track. Overall, FCVA-deposited COC without and with lube (samples S-3 and S-9) showed remarkable tribological properties, which is very important for developing ultrathin yet protective coatings for magnetic media.

## Discussion

In order to enhance the durability and to achieve high areal density hard disk drives with good signal to noise ratio, possessing lower friction, higher wear resistance, higher oxidation/corrosion resistance and lower surface energy are amongst the desirable characteristics from COCs at lower thicknesses. The analysis of functional properties revealed that all these characteristics were found to be better in media with ~1.7 nm COC deposited at 350 eV followed by 90 eV using FCVA (sample S-3) as compared to media with thicker ~2.7 nm commercial COC (sample S-2). To understand the cause of the improved functional properties in COC of sample S-3 than sample S-2, microstructural and morphological analyses were performed. The HRTEM, STM, mass spectrometry and Raman analyses suggested that unlike the commercial COC, the COC in sample S-3 seems to contain some amount of local interspersed graphene/fullerene-like nanostructures within the amorphous matrix of carbon. In addition, XPS analysis revealed that the FCVA deposited COC in sample S-3 possesses higher sp^3^ C-C bonding than commercial COC in sample S-2. Hence, local interspersion of graphene/fullerene-like nanostructures within the amorphous matrix of carbon and higher sp^3^ bonding seem to be the cause of improved surface and functional performance of FCVA deposited COC in sample S-3. It is interesting to note that the tribological and surface properties of media with ~1.7 nm COC deposited using FCVA was further improved when a lubricant layer was deposited atop it (S-9). On the other hand, full commercial media with ~2.7 nm COC and ~1 nm lubricant layer showed almost similar properties with its non-lubricated counterpart, except for its surface energy. We shall elaborate our discussion to understand the mechanisms of 1) formation of the local dispersion of graphene/fullerene-like nanostructures within amorphous carbon matrix, 2) formation of higher sp^3^ C-C bonding in COC of sample S-3, 3) the structure-property relationship for different samples and 4) how application of lubricant further improves the proeprties of media with COCs.

Usually, the FCVA and PECVD processes produce amorphous carbon films^3^. In fact, the FCVA process forms a tetrahedral amorphous carbon (ta-C) film, which is a highly disordered form of carbon^3^. Under special process conditions during film growth such as high pressure and high temperature, these methods can produce nanostructure-embedded carbon films. Interestingly, we observed the formation of carbon film with such a nanostructure by FCVA without using any specific process conditions that is at room temperature with usual deposition pressure (~2.5 × 10^-6^ Torr), although we employed a novel idea of bi-level surface modification of media using dual energies. The formation of such nanostructures in carbon is very interesting and the possible underlying science is proposed below. In our case, three possible factors which could assist to obtain nanostructures within carbon films by FCVA are: 1) the energetics of the process, 2) the presence of a metallic substrate, and 3) very low carbon thickness. We have deposited a ~1.7 nm carbon film, which comprises only a few atomic layers, onto the CoCrPt based media using dual C^+^ ion energies of 350 and 90 eV by FCVA. Co and Pt are transition metals and actively participate in catalytic activities. Co and Pt are used as catalysts to grow low dimensional carbon materials such as graphene^51^,^52^ and carbon nanotubes^26^,^53^ at higher temperatures. In our case, we expect that when COC is deposited onto the CoCrPt-alloy based media substrate; the metallic Co and Pt atoms present in the media would catalyze the COC. The catalytic nature of Co and Pt at room temperature growth (no intentional heating) arises due to the energetics of the FCVA deposition process. When carbon ions with energies of 350 eV and 90 eV strike the substrate, they may raise the local temperature considerably (referred to as localized heating) of the substrate surface. Weismental *et al.*^54^ suggested that for C^+^ ions with energy of 100 eV, a region with a radius of ~0.75 nm can be heated up to a local temperature of ~3823 K. Hence, with energy of 350 eV, the heating temperature and the heated region may vary or even become higher and larger than that achieved at 100 eV. Since the thickness of COC comprises only few carbon layers, the catalytic effect of Co and Pt on the carbon may be substantial due to closer proximity of the media to the topmost COC surface, producing a larger active carbon region. As a consequence, this can lead to the formation of graphene-like nanostructures in the localized region of the amorphous carbon, as evidenced by STM and HRTEM. Now we discuss on the possible factors causing the formation of fullerene-like structures in amorphous carbon matrix. The formation of either fullerene-like carbon films or fullerene-like nanostructures in amorphous carbon has been demonstrated by either using nitrogen in case of sputtering or creating a high localized pressure during the FCVA process. The transformation of carbon to fullerene-like onions by the metal-based catalyst approach has also been reported^55^. The whole concept is directed to either achieve a desired total energy or reduce the cost of total energy required to curve the basal planes. Since sputtering is not an energetic process, the available energy is not high enough to curve the basal planes and obtain pentagons in the pure carbon structure^23^. Based on total energy calculations, Sjostrom *et al.*^21^ had suggested that an energy of ~73.8 kcal/mol is required to form pentagons in a sheet structure. However, they suggested that if two carbon atoms in pentagons are substituted by nitrogen atoms the energy cost can be reduced by ~26.2 kcal/mol. This explains why the incorporation of nitrogen during sputtering of carbon films can promote the formation of either fullerene-like carbon films or fullerene-like nanostructures in carbon films. On the other hand, Amaratunga *et al.*^14^ had demonstrated the formation of amorphous carbon films by vacuum arc, which contained embedded fullerene-like onion nanostructures. The system which they had used for the deposition of their films was the same as the one which was used in this work except for one additional modification. During the vacuum arc growth process, they had created a high local gas pressure to produce fullerene-like onion nanostructures embedded amorphous carbon films^14^. In our case, a bi-level surface modification was performed where the energetics, the metal-based catalyzation and the low carbon film thickness all assist to generate the fullerene-like nanostructures within the local regions of the amorphous carbon. These conditions may therefore be favorable to either achieve desired energy or reduce the cost of energy to form fullerene-like nanostructures in an amorphous carbon matrix. The role of energetics in the formation of graphene/fullerene-like nanostructures within the carbon film can be seen by comparing the film deposited by the bi-level surface modification scheme at 350/90 eV with the carbon films deposited at 20–25 eV using FCVA. The films deposited at 20–25 eV and with thicknesses of 1.2 nm and 1.6 nm (samples S-4 and S-5) displayed the formation of carbon nanoaggregates (Fig. 4a–f), which was also seen in the PECVD-based commercial COC in sample S-2 (Fig. 3g–i). The morphology of carbon remained similar, when low energy bi-level modification was performed at 90 eV, followed by 50 eV (Fig. 4g–i). This indicates that a greater contribution from the ion energetics, which can be supplied by increasing the deposition ion energy, is required to transform the nanoaggregates-type morphology/topography into graphene/fullerene-like nanostructures within amorphous carbon. This explains why the amorphous carbon film with such nanostructures was formed due to the use of slightly higher ion energy of 350 eV followed by 90 eV ion energy in our FCVA bi-level surface modification process.

We now explain the mechanism of formation of higher sp^3^ bonding in the COC of sample S-3. The observed higher sp^3^C fractions in the FCVA-processed COC, even at the ultrathin regime, are due to the following reasons. FCVA generates highly ionized plasma (~90 %) of C^+^ ions. The energetics of the C^+^ ions can be controlled by varying the negative substrate bias. When the energy of the C^+^ ions is high enough, they penetrate the outermost atomic layer of the growing carbon film, leading to subsurface growth. This subsurface growth leads to increased density and hence enhances the sp^3^ bonding in the carbon film. This subsurface growth model is known as subplantation^3^,^56^. Moreover, the optimum ion energy to grow a highly sp^3^ bonded carbon film is 90–100 eV^3^. In the present case, rather than using a single ion energy, we have employed two different energies (initially 350 eV followed by 90 eV) for growing COC on media. The C^+^ ions at 350 eV cause atomic mixing of C with the Co, Cr and Pt atoms in the magnetic media layer. Subsequently, the C^+^ 90 eV ions form a highly sp^3^ bonded carbon layer. This approach helps in enhancing the adhesion of COC with the underlying magnetic media due to atomic mixing and at the same time provides higher sp^3^C bonding and a dense carbon layer due to the use of the optimum ion energy of 90 eV.

We now move over to the discussion on structure-property relationship. The contact angle measurements revealed that media with FCVA-deposited COC (sample S-3) showed significantly higher water contact angle and lower surface energy than commercial COC (sample S-2). The observed difference in contact angle and surface energy can be explained in view of chemical composition and geometric structure (roughness) of the overcoated media surfaces. Since roughness of these samples is very low to have its influence on contact angle, we negate this factor for contact angle measurement and concentrate on the chemical compositional factor for explaining the results. The commercial COC in sample S-2 is a bilayer overcoat with hydrogenated carbon (CH_x_) as a main layer followed by a very thin layer of nitrogenated carbon^57^. On the other hand, FCVA-deposited film is delibretaly grown as a non-hydrogenated and non-nitrogenated carbon (pure carbon layer). As shown, the structure of FCVA-deposited COC consists of sp^3^ rich amorphous carbon with local interspersion of graphene/fullerene-like nanostructures. One of the reasons for the lower water contact angle in media with commercial COC (sample S-2) could be the strong interaction of water with hydrogen atoms available in CH_x_ overcoat, leading to greater physisorption of water to the surface and hence, higher wetting behaviour was observed in commercial COC. On the other hand, FCVA-deposited COC does not possess hydrogen in its structure, giving rise to more hydrophobic behaviour. Further, since carbon-oxygen bonds are polar becuase of difference in their electronegativity, the amount of carbon-oxygen bonding can also influence the hydrophobic behaviour of carbon^58^. The XPS analysis revealed that amount of carbon-oxygen bonds (C-O and C=O) are more in commercial COC than FCVA deposited COC (*see suppelementary information S4*), lower carbon-oxygen bonding seems to be advantageous for enhancing the hydrophobic behaviour of material. Apart from them, the interesting factor in explaning the excellent hydrophobic behaviour of FCVA-deposited COC is the presence of localized graphene/fullerene-like nanostructures in amorphous carbon matrix as they exhibit highly hydrophobic behaviour^58^,^59^,^60^,^61^,^62^.

The hydrophobicity of media with both types of COCs was enhanced and surface energy was decreased when PFPE-based lubricant was deposited on them. PFPE is a fluorocarbon based fluoropolymer and has backbone of a linear copolymer of tetrafluoroethylene-oxide and difluoromethylene-oxide with presence of –OH end groups, structure of which is shown below.




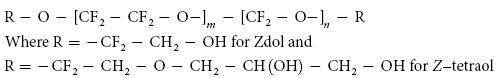




In PFPE, presence of OH end groups is assisted to enhance the bonding of lubricant with COC. Since fluorocarbon shows highly hydrophobic behaviour and lower surface energy, the improved hydrophobic behaviour and reduced surface energy after depositon of PFPE lubricant in samples S-8 and S-9 is attributed to the presence of tetrafluoroethylene-oxide and difluoromethylene-oxide. However, between two samples S-8 and S-9, slightly higher hydrophobic behaviour and lower surface energy was obtained in sample S-9. This could be due to the following two facts: 1) When PFPE lubricant is deposited onto the CH_x_ based commercial COC, some fraction of fluroine atoms would participate by interacting with the H-atoms of CH_x_ overcoat via strong hydrogen bonding. On the other hand, since FCVA-deposited COC is non-hydrogenated, almost all of fluorine atoms would be present in its original state within the PFPE and contributes towards enhancing the hydrophobicity. This explains why significantly higher (lower) hydrophocity (surface energy) was found in sample S-9 than sample S-8. 2) The hydrophocity (surface energy) was significantly higher (lower) in sample S-3 than sample S-2. Hence, base level, which is S-3 for S-9 and S-2 for S-8, can contribute in observing slightly better surface properties in sample S-9 than sample S-8.

Further, from results of tribological properties, it was observed that FCVA-deposited COC in sample S-3 showed very stable and lower friction and higher wear-resistance than commercial COC in sample S-2. The reasons for such exceptional tribological characteristics of the FCVA-processed COC are threefold: 1) The COC in sample S-3 was deposited using the bi-level surface modification scheme. Initial bombardment at 350 eV led to atomic mixing, which enhances adhesion of the COC with media. Subsequently, bombardment at 90 eV helped to form a dense and high sp^3^C bonded layer. Thus, both atomic mixing and high sp^3^C bonding helped to acheive low and stable COF and high wear resistance. 2) The RMS surface roughness of sample S-3 was lower than that of sample S-2 (~0.2 nm versus ~0.5 nm), as obtained by STM measurements, which could have influenced the tribological proeprties in terms of lower COF. 3) Most importantly, COC in sample S-3 contain interspersed graphene/fullerene-like nanostructures in an amorphous carbon matrix, which help in providing the rigidity, elasticity and hence, higher wear resistance to the coating and offer lower friction^11^,^12^,^25^,^63^,^64^. The tribological properties of FCVA-deposited COCs were enahnced after the depositon of lubricant in terms of lower and stable friction. Samad *et al.*^6^ have demonstrated the improvement in tribological properties of carbon embedded cobalt after the deposition of PFPE lubricant. Similar characteristics were also observed by Chen *et al.*^65^, who demonstrated the improved tribological properties of carbon coated Al_2_O_3_-TiC sliders after the deposition of ZDOL lubricant.

In conclusion, we have presented a method of synthesizing ultrathin COCs with interspersed graphene/fullerene-like nanostructures on CoCrPt-based media using the FCVA process, and have compared its functional performance with current commercial media having a thicker COC. Raman spectroscopy, TOF-SIMS, STM and HRTEM analyses confirmed the presence of the interspersed graphene/fullerene-like nanostructures in the amorphous carbon matrix of the ultrathin COC formed using the FCVA process. A fundamental understanding was developed on the formation of these nanostructures in COC based on the energetics of the process and the catalytic activities of Co and Pt. In addition, the FCVA-processed COC was found to show higher sp^3^ carbon bonding than current commercial COC despite a reduction of ~37 % in its thickness. The evaluation of its functional performance in terms of tribology, surface properties and oxidation/corrosion resistance revealed the superiority of FCVA-processed COC over current commercial COC. The thinner (~1.7 nm) FCVA- processed COC showed lower friction, higher wear resistance, higher hydrophobic behaviour, lower surface energy and comparable/better corrosion and oxidation protection than thicker (~2.7 nm) current commercial COC. The improved characteristics in FCVA-processed COC can be attributed to the combined effects of the formation of the unique graphene/fullerene-like nanostructures, higher sp^3^ carbon bonding and lower surface roughness. After lubrication, the tribological proeprties of the FCVA-deposited COC was further improved due to the synergetic effect of the graphene/fullerene-like nanostructure embedded COC and the lubricant. These findings are extremely important for the development of ultrathin (<2 nm) yet protective COCs for future hard disk media.

## Methods

### Samples preparation

We have compared functional performance of mainly three different types of samples in this work. However, to understand the role of energetics on the fabrication of nanostructures embedded COCs, three other samples were also synthesized. Sample S-1 was a specially prepared CoCrPt:oxide bare magnetic media disk without COC and lubricant (lube) obtained from our industry collaborators, which was then plasma etched for fair comparison. The plasma etching was performed in a magnetron sputtering system (AJA Inc.) using Ar plasma at a base pressure of 2 × 10^−8^ Torr, working pressure of 1.0 × 10^−2^ Torr, Ar gas flow rate of 20 sccm, and a substrate RF power of 40 W for 3 min. Sample S-2 was a specially prepared CoCrPt:oxide current commercial media disk with commercial COC of thickness ~2.7 nm but with no lube. Sample S-3 was a FCVA-processed COC deposited at dual C^+^ ion energies of 350 eV followed by 90 eV (350/90 eV). The respective ion energies were achieved by supplying a pulsed substrate bias of −330 V in the first stage and −70 V in the second stage of the bi-level surface modification using the FCVA process, with a frequency of 20 kHz and a duty cycle of 60% (each cycle consisted of 30 μs of applied bias, followed by 20 μs of no bias). During the deposition of COC in sample S-3, the pressure was ~2.5 × 10^−6^ Torr and the temperature was room temperature. The starting substrate for the preparation of sample S-3 was CoCrPt:oxide based current commercial hard disk media disk with its origninal COC and lube, which were then removed before deposition of the FCVA processed COC (as explained in results and discussion section). In addition, to study the role of energetics on the fabrication of graphene/fullerene-like nanostructures embedded COCs, three other samples were also prepared at relatively lower ion energies and examined by scanning tunneling microscopy (STM). Out of these three samples, two of them have ultrathin COCs of thicknesses ~1.2 nm (sample S-4) and ~1.6 nm (sample S-5) and were deposited at C^+^ ion energy of 20–25 eV by FCVA. A sixth sample was formed under low energy bi-level surface modification using FCVA. The low energy bi-level surface modification was conducted at 90 eV followed by 50 eV (90/50 eV, sample S-6). The thickness of sample S-6 was measured to be ~1.9 nm. The substrate and the deposition strategies for the deposition of these three samples (S-4, S-5 and S-6) were kept similar to that of sample S-3.

### Lubricant coating

Perflurouropolyether (PFPE) lubricant was used in this study to understand the role of lubricant on the tribological properties of bare and COC-containing media. After cleaning, samples S-1 and S-3 were dip-coated with PFPE (0.08 wt% Zdol 4000 in HFE 7100 solvent) using a dip coating tool situated in our clean room (class 10 K). The samples were immersed and withdrawn at a speed of 0.5 mm/s. Before withdrawing, the samples were held in the PFPE solution for 20 s. After the coatings have been done, the PFPE-coated samples were cured at a temperature of 150 °C for 1.5 h to improve the bonding of the lubricant with the underlying surface. After discussing with our industrial collaborators and based on our optimization, the process parameters and other conditions were maintained so as to obtain ~1–1.2 nm lubricant thickness. After the deposition of PFPE lubricant onto samples S-1 and S-3, the lubricated samples are named as S-7 and S-9, respectively. In addition, we have included the full commercial media with ~2.7 nm commercial COC and ~1 nm commercial lubricant (sample S-8) in this study in order to compare the tribological properties of our complete media and current commercial media.

### Characterizations

High resolution transmission electron microscopy (HRTEM: Philips CM300 FEG) in cross-section geometry was employed to determine the thicknesses of COCs in samples S-2 and S-3 on hard disk media. Before capturing the HRTEM images, samples were prepared in various steps: first, the deposition of a capping layer for image contrast, followed by mechanical grinding/polishing to thin down the sample and finally, ion milling. STM measurements were performed in ultra-high vacuum (UHV) conditions (~4 × 10^−11^ mbar) and at a temperature of 77.8 K using a tungsten tip by UHV Omicron LT STM system. Time of flight secondary ion mass spectrometry (TOF-SIMS, ON TOF Gmbh, Germany) and Raman spectroscopy measurements (Jobin Yvon LABRAM-HR) were carried out to examine the molecular structure and microstructure of carbon in samples S-2 and S-3. TOF-SIMS measurements were conducted under UHV conditions, where pulsed primary ions from a 25 keV Bi liquid?metal ion gun (LMIG) were used to bombard the sample surface to generate the secondary ions. Raman measurements were performed using a visible excitation wavelength of 488 nm from Ar laser, while keeping a spot size of ~1 μm for the analysis. To avoid excessive laser heating on such ultrathin layers, the excitation laser power was kept very low. Surface chemical analyses on the samples were carried out using X-ray photoelectron spectroscopy (XPS) in an UHV condition of ~3 × 10^−9^ Torr with a monochromic Al K_α_ X-ray source employing a spot size of ~400 μm for analysis. Ball-on-disk tribological tests were performed in a clean room area (temperature ~25 °C and relative humidity ~55%) using a CSM-Nanotribometer (Switzerland). A sapphire ball of 2 mm diameter and surface roughness of 5 nm was used as the counterface material. Before starting each test, the ball was carefully cleaned using acetone and immediately imaged using an optical microscope to avoid any contamination from the ball surface. During the tests, the wear track radius was fixed at 2 mm and the normal load was fixed at 20 mN. The measurements were performed at a constant rotational speed of 100 rpm (linear speed of 0.021 m/s) for 10000 cycles. After each test, the sample surface and the ball were imaged using an optical microscope to observe any material transfer or wear on the surfaces.

Surface energy of the samples was measured using the combination of contact angles of two liquids—water and diiodomethane. VCA Optima (AST Product Inc.) goniometer was used to measure the contact angles at room temperature. The size of droplets was fixed to 0.5 μl in each measurement and the angles were measured 10 s after the droplets were formed on the sample surfaces once being expelled from the needle. The reason for delaying 10 s was to give enough time for the interaction of droplets with the solid surfaces. A total of five measurements were performed on each sample for repeatability and accuracy.

## Additional Information

**How to cite this article**: Dwivedi, N. *et al.* Ultrathin Carbon with Interspersed Graphene/Fullerene-like Nanostructures: A Durable Protective Overcoat for High Density Magnetic Storage. *Sci. Rep.* **5**, 11607; doi: 10.1038/srep11607 (2015).

## Supplementary Material

Supplementary Information
