# Supplementary material for: Ultrathin Carbon with Interspersed Graphene/Fullerene-like Nanostructures: A Durable Protective Overcoat for High Density Magnetic Storage
Source: Sci Rep. 2015 Jun 25;5:11607. doi: 10.1038/srep11607 (PMC4479824; doi:10.1038/srep11607)
Supplement: Supplementary Information [file srep11607-s1.pdf]

Supporting Information

Ultrathin Carbon with Interspersed Graphene/Fullerene-like Nanostructures: A Durable Protective Overcoat for High Density Magnetic Storage

Neeraj Dwivedi[a], Nalam Satyanarayana[a], Reuben J. Yeo[a], Hai Xu[b], Kian Ping Loh[b], Sudhiranjan Tripathy[c], Charanjit S. Bhatia[a,*]

[a]Department of Electrical and Computer Engineering, National University of Singapore, Singapore 117583

[b]Graphene Research Centre and Department of Chemistry, National University of Singapore Singapore 117543

[c]Institute of Materials Research and Engineering (IMRE), A*STAR (Agency for Science, Technology, and Research), 3 Research Link, Singapore 117602

***Corresponding Author**

Email: elebcs@nus.edu.sg (C. S. B.)

Tel: +65-6516 7216

S1. Samples Description and Nomenclature

Table S1: Description and nomenclature of the samples used in this work.

Samples	Nomenclature and Description
**S-1**	Plasma cleaned specially prepared CoCrPt-oxide based commercial media with no COC and no lubricant
**S-2**	Specially prepared CoCrPt-oxide based commercial media with ~ 2.7 nm commercial COC but no lubricant
**S-3**	FCVA-deposited ~ 1.7 nm COC on CoCrPt-oxide based media. The deposition was carried out at ion energy of 350 eV followed by 90 eV.
**S-4**	FCVA-deposited ~ 1.2 nm COC on CoCrPt-oxide based media. The deposition was carried out at ion energy of 20-25 eV.
**S-5**	FCVA-deposited ~ 1.6 nm COC on CoCrPt-oxide based media. The deposition was carried out at ion energy of 20-25 eV.
**S-6**	FCVA-deposited ~ 1.9 nm COC on CoCrPt-oxide based media. The deposition was carried out at ion energy of 90 eV followed by 50 eV.
**S-7**	Plasma cleaned specially prepared CoCrPt-oxide based commercial media with no COC but with in-house deposited ~ 1.4 nm thick ZDOL lubricant.
**S-8**	Specially prepared CoCrPt-oxide based commercial media with ~ 2.7 nm commercial COC and ~ 1 nm PFPE based lubricant.
**S-9**	FCVA-deposited ~ 1.7 nm COC on CoCrPt-oxide based media. The deposition was carried out at ion energy of 350 eV followed by 90 eV. After COC deposition ~ 1.4 nm thick ZDOL lubricant was applied on media containing COC using dip-coating process.

S2. STM Analysis

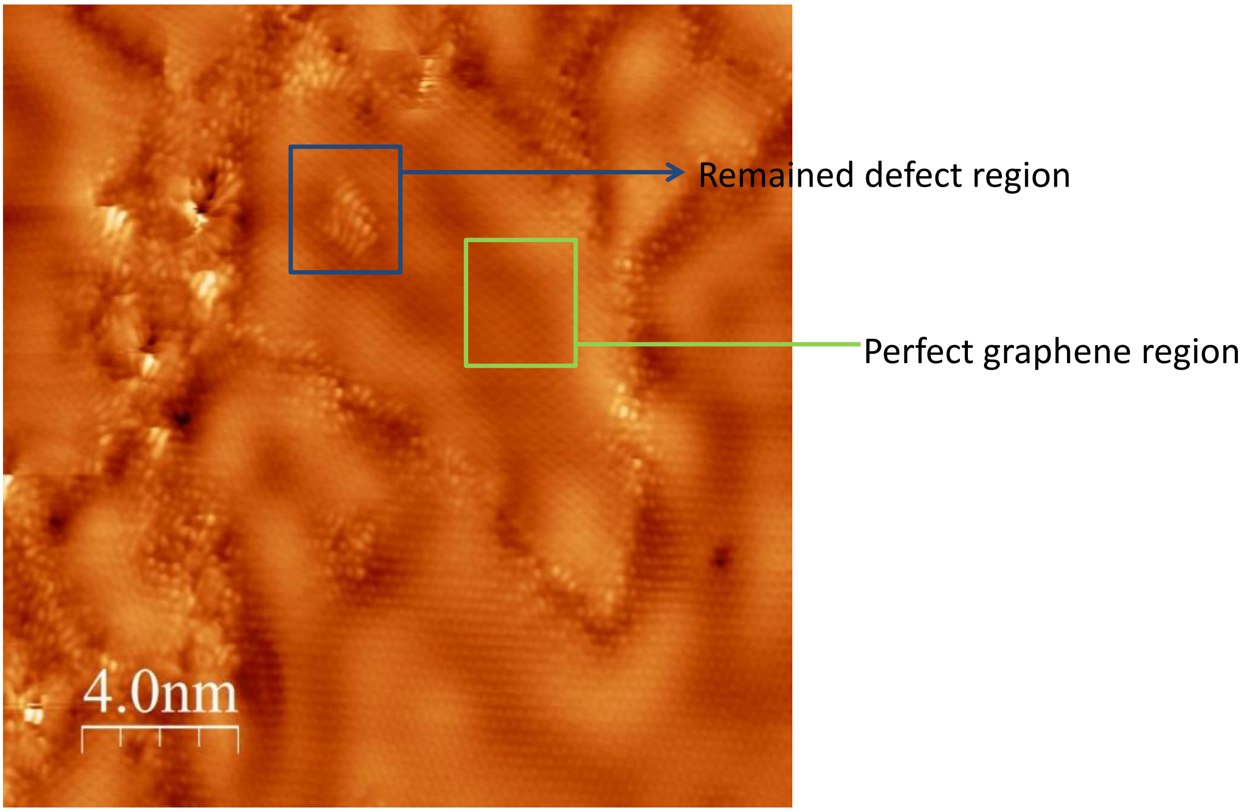

Figure S2: Synthesis of graphene on Cu 111.

Figure S2 shows the formation of graphene on Cu 111 substrate. It is clear from the Figure that some parts of the sample showed formation of perfect graphene, while some parts of the sample revealed the formation of defected graphene. The morphology of defected graphene appears to be very similar to the STM image of COC in sample S-3 (Fig. 3b of main manuscript).

S3. Raman Analysis

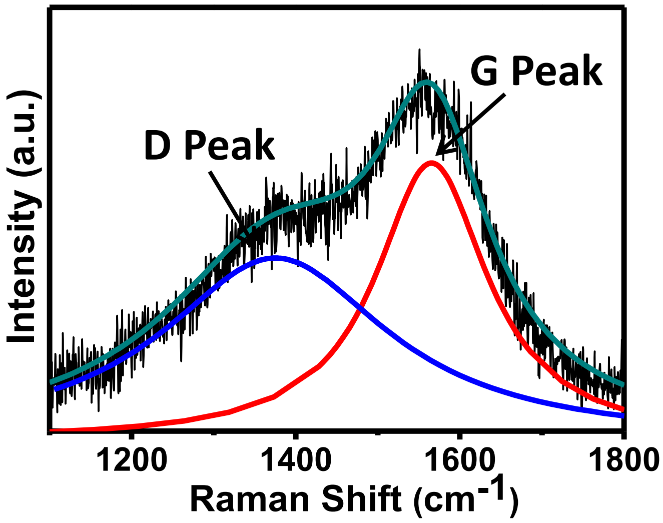

Figure S3: Fitted Raman spectra of sample S-2. G peak was fitted using BWF function while D was fitted using Lorentzian function.

S4. ARXPS Analysis

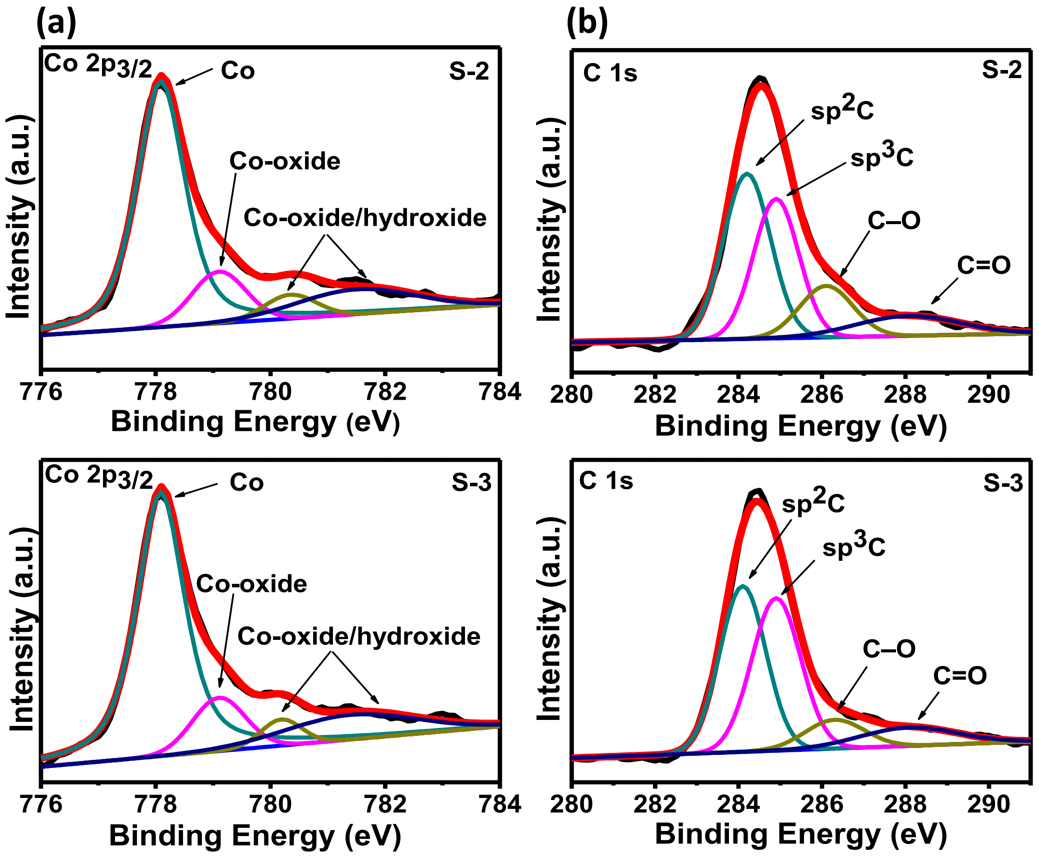

Figure S4.1: Deconvoluted (a) Co $2p_{3/2}$ and (b) C 1s core level spectra of sample S-2 and S-3.

Table S4.2: Quantitative analysis of various bonds examined from C1s and Co $2p_{3/2}$ core level spectra.

Samples	Core level	Binding energies (B.E.) and % of constituent peaks				
S-2	C 1s		sp^2C	sp^3C	C–O	C=O
		B.E. (eV)	284.2	284.9	286.1	288.0
		fraction %	41.5	33.0	14.5	11.0
	Co $2p_{3/2}$		Co	Co-oxide	Co-oxide/hydroxide	
		B.E. (eV)	778.1	779.1	780.3	781.5
		fraction %	67.9	12.9	5.7	13.5
S-3	C 1s		sp^2C	sp^3C	C–O	C=O
		B.E. (eV)	284.1	284.9	286.3	2881.
		fraction %	41	40	9	10
	Co $2p_{3/2}$		Co	Co-oxide	Co-oxide/hydroxide	
		B.E. (eV)	778.1	779.1	780.2	781.4
		fraction %	70	12	4.5	13.5

S5. Surface Energy Measurements

Contact angle measurements with two liquids — water and diiodomethane — were performed to understand the hydrophobic behaviour and to estimate the various components of surface energies. The results and discussion are given in main manuscript but the theory and formula used for estimating them are described here. The Good-Girifalco-Fowkes-Young method was used to calculate the respective surface energies of these samples[1-3]. Young's equation for the equilibrium of a liquid drop under the action of three surface tensions is given by:

$$\gamma_{sv} = \gamma_{sl} + \gamma_{lv}\cos\theta, \qquad (1)$$

where γ_{sv}, γ_{lv} and γ_{sl} are the surface tensions at the solid-vapor, liquid-vapor and solid-liquid interfaces respectively, and θ is the contact angle of the liquid on the solid surface.

Fowkes proposed that the dispersion interaction between two phases such as solid and liquid, L_{sl}^{d}, can be given by the geometric mean of the individual dispersion components of two phases (for example, solid, L_{sv}^{d}, and liquid, L_{lv}^{d}) as:

$$\mathrm{L}_{sl}^{d} = \sqrt{\mathrm{L}_{sv}^{d}\mathrm{L}_{lv}^{d}} = 2\sqrt{\gamma_{sv}^{d}\gamma_{lv}^{d}} \quad (2)$$

Since a non-polar liquid with only dispersion surface free energy (γ_{lv}^{d}) interacts with only the dispersion component of the solid, the dispersion component of the surface free energy of the solid can be estimated from the contact angle of the non-polar liquid:

$$\gamma_{sv}^{d} = \frac{\gamma_{lv}^{d}(1+\cos\theta)^2}{4}, \quad (3)$$

Similar to the dispersion interaction, the polar interaction between two phases such as solid and liquid, L_{sl}^{p}, can be given by the geometric mean of the individual dispersion components of two phases (solid, L_{sv}^{p}, and liquid, L_{lv}^{p}) as:

$$\mathrm{L}_{sl}^{p} = \sqrt{\mathrm{L}_{sv}^{p}\mathrm{L}_{lv}^{p}} = 2\sqrt{\gamma_{sv}^{p}\gamma_{lv}^{p}} \quad (4)$$

According to Berthelot, the work of adhesion between a solid and a liquid can be estimated as the geometric mean of the cohesive works of the solid and liquid as:

$$W_{sl} = \sqrt{W_{ss}W_{ll}} = 2\sqrt{\gamma_{sv}\gamma_{lv}} = \gamma_{sv} + \gamma_{lv} - \gamma_{sl} = \gamma_{lv}(1+\cos\theta), \quad (5)$$

The liquid with both dispersion (γ_{lv}^{d}) and polar components (γ_{lv}^{p}) interacts with both the dispersion and polar components of the solid surface and follows the relation:

$$\gamma_{lv}(1+\cos\theta) = \mathrm{L}_{sl}^{d} + \mathrm{L}_{\mathrm{sl}}^{\mathrm{P}} \quad (6)$$

Using equations (2) and (4), equation (6) can be re-written as:

$$\gamma_{lv}(1+\cos\theta) = 2\sqrt{\gamma_{sv}^{d}\gamma_{lv}^{d}} + 2\sqrt{\gamma_{sv}^{p}\gamma_{lv}^{p}}, \quad (7)$$

Since all the other parameters are known, the polar surface free energy of solid,γ_{sv}^{p}, can be estimated from equation (7).

Employing equations (3) and (7), we calculated the dispersion, polar and total surface energies of solid for the samples of interest, which are presented in Figure 7 of the main manuscript.

References

1. Good, R. J. & Girifalco, L. A. A theory for estimation of surface and interfacial energies.III Estimation of surface energies of solids from contact angle data. *J. Phys. Chem.* **64**, 561 (1960).
2. Fowkes, F. M. Determination of interfacial tensions, contact angles and dispersion forces in surfaces by assuming additivity of intermolecular interactions in surfaces. *J. Phys*. Chem. **66**, 382 (1962).
3. Van Oss, C. J., Chaudhury, M. K & Good, R. J. Interfacial Lifshitz-van der Waals and polar interactions in macroscopic systems. *Chem. Rev.* **88**, 927 (1988).
